# Feasibility of Ultra-Thin Fiber-Optic Dosimeters for Radiotherapy Dosimetry

**DOI:** 10.3390/s151129003

**Published:** 2015-11-17

**Authors:** Bongsoo Lee, Guwon Kwon, Sang Hun Shin, Jaeseok Kim, Wook Jae Yoo, Young Hoon Ji, Kyoung Won Jang

**Affiliations:** 1 School of Biomedical Engineering, College of Biomedical & Health Science, BK21 Plus Research Institute of Biomedical Engineering, Konkuk University, 322 Danwol-dong, Chungju-si, Chungcheongbuk-do 380-701, Korea; E-Mails: bslee@kku.ac.kr (B.L.); lucky7568@naver.com (G.K); shshin9431@gmail.com (S.H.S.); kimzgo@naver.com (J.K.); wonzip@naver.com (W.J.Y.); 2 Research Center for Radiotherapy, Korea Institute of Radiological and Medical Sciences, 215-4, Gongneung-dong, Nowon-gu, Seoul 139-706, Korea; E-Mail: jyh328@kirams.re.kr

**Keywords:** fiber-optic sensors, radiation dosimeter, Co-60, radiation therapy

## Abstract

In this study, prototype ultra-thin fiber-optic dosimeters were fabricated using organic scintillators, wavelength shifting fibers, and plastic optical fibers. The sensor probes of the ultra-thin fiber-optic dosimeters consisted of very thin organic scintillators with thicknesses of 100, 150 and 200 μm. These types of sensors cannot only be used to measure skin or surface doses but also provide depth dose measurements with high spatial resolution. With the ultra-thin fiber-optic dosimeters, surface doses for gamma rays generated from a Co-60 therapy machine were measured. Additionally, percentage depth doses in the build-up regions were obtained by using the ultra-thin fiber-optic dosimeters, and the results were compared with those of external beam therapy films and a conventional fiber-optic dosimeter.

## 1. Introduction

The purpose of radiation therapy is to deliver suitable doses to lesions and to minimize the doses to normal tissues simultaneously. Thus, it is important to measure the actual doses delivered to the normal tissues to prevent side effects. In the normal tissues, especially, skin is composed of the epidermis, dermis and hypodermis [1]. The basal layer, which is the innermost layer of the epidermis, is very vulnerable to ionizing radiations; here, the radiation induces injuries including erythema, desquamation, and necrosis on and in the skin [2]. However, measurement of the skin dose is very difficult with conventional radiation dosimeters such as ionization chambers, thermoluminescence dosimeters (TLDs) and external beam therapy (EBT) films. Ionization chambers have commonly been used in radiotherapy dosimetry, but they have several drawbacks such as complicated calibration process and non-water equivalency [3,4]. Although TLDs and EBT films can be used to measure the skin dose, these types of dosimeters lack the ability for real-time measurements and require a time-consuming readout procedure [5,6]. 

Meanwhile, fiber-optic dosimeters (FODs) have been developed for radiotherapy dosimetry [7,8,9]. Generally, FODs consist of organic scintillators, plastic optical fibers (POFs), and photodetectors [10,11]. As sensing probes of the FODs, organic scintillators emitting scintillations proportional to the absorbed dose causes minimal dosimetric perturbations in a plastic or water phantom by virtue of their small sizes (usually 0.5–1.0 mm) [12,13]. In addition, both of the scintillators and the fibers consist of nearly water-equivalent materials; this enables us to avoid complex conversions arising from the material difference between a dosimeter and a tissue. Unfortunately, although FODs have favorable characteristics for radiotherapy dosimetry, diameters or thicknesses of typical FODs are significantly too large to measure skin doses. Practically, in our previous study, the measured surface doses using the FOD incorporating a cylinder-type organic scintillator with 0.5 mm diameter were higher than the results of EBT films due to relatively large sensing volume [14]. The International Commission on Radiological Protection (ICRP) recommends assessing the skin dose for the basal layer at a depth of 70 μm [15]. In order to measure the skin dose accurately, therefore, the dosimeter should have a sensing probe shaped like a pellicle with a water equivalent material. 

To solve the above problems, we propose a new approach to skin dosimetry using ultra-thin fiber-optic dosimeters (UTFODs). In this study, prototype UTFODs were fabricated with organic scintillators, wavelength shifting fibers (WSFs), and POFs. The sensor probes of the UTFODs consist of very thin organic scintillators with thicknesses of 100, 150 and 200 μm; these are significantly small relative to the organic scintillator used in our previous study. These types of sensors cannot only be used to measure the skin or surface doses but also provide depth dose measurements with higher spatial resolution in comparison with the conventional FODs. To evaluate the feasibility of the UTFODs, the percentage depth doses (PDDs) in a build-up region of gamma-ray beams from a Co-60 therapy machine were measured with the UTFODs, and we compared the results with those of EBT films and a conventional FOD. Additionally, surface doses were measured according to the thicknesses of the scintillators and irradiation field sizes. Finally, we evaluated the angular and linear responses of the UTFODs to the irradiation angles and the absorbed doses, respectively.

## 2. Materials and Methods

Throughout the study, organic scintillators (BCF-12, Saint-Gobain Ceramic and Plastics) were used as a sensing material. The scintillator has a core/clad structure and its diameter is 1.0 mm. The thickness of the cladding is about 4% of the outer diameter. The core and the cladding are composed of the polystyrene (PS) and the polymethyl methacrylate (PMMA), respectively. The physical properties of the BCF-series used in this study are presented in Table 1. The square-type WSFs (BCF-92, Saint-Gobain Ceramic and Plastics) were also used to increase the collection efficiency of the scintillations emitted from the scintillators. The length and thickness of the WSF are 10 mm and 1.0 mm, respectively, and other physical properties are listed in Table 1.

**Table 1:** Properties of the BCF-series used in the experiment.

	BCF-12	BCF-92	BCF-98
Emission peak	435 nm	492 nm	-
Decay time	2.7 ns	3.2 ns	-
Refractive index	1.60 (core), 1.49 (cladding)		
Density	1.05 g/cm^3^		
Numerical aperture (NA)	0.58		

To transmit the light signals generated from UTFODs to a photo detector, multi-mode plastic optical fibers (GH-4001, Mitsubishi) with a diameter of 1.0 mm were exploited. The core and the cladding have the refractive indices of 1.492 and 1.402, respectively, and the numerical aperture (NA) is 0.5. The core is made of PMMA, and the cladding is fluorinated polymer.

**Figure 1:**
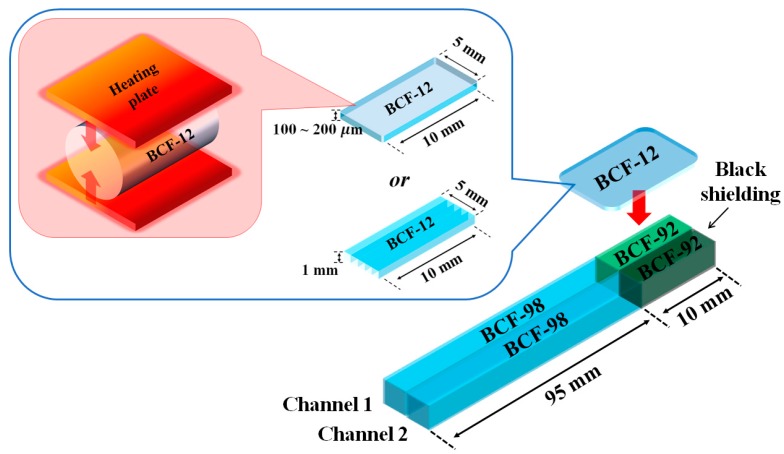
Manufacturing process and structure of ultra-thin fiber-optic dosimeters (UTFODs).

The manufacturing process and structure of the UTFODs are shown in Figure 1. In this study, scintillators with 100 ± 11, 150 ± 8, and 200 ± 13 μm thicknesses were fabricated by pressing 1.0-mm-diameter organic scintillators (BCF-12) with a heating plate. Since the high temperature (about 200 °C) is treated on the organic scintillators, the scintillation properties of the scintillators can vary after the fabrication process. In order to clarify the heating effect on the scintillation properties, the scintillation yields of two BCF-12s treated respectively with and without the heating process (about 200 °C) were measured. Because the geometry of BCF-12 varies after the heating, a stainless steel based cylindrical frame was exploited. Also, both the lengths of BCF-12s were fixed as 1.0 cm. In this experiment, the scintillation yield of BCF-12 treated with the heating decreased by 12% in comparison to that without the heating; this phenomenon can be induced by the geometrical structure and optical property changes of the scintillator. The square-type scintillator array was also used for the conventional FOD; here, 5 commercial grade 1.0 mm-thick BCF-12s which were not treated with the heating and pressure were exploited. The UTFODs fabricated in this study consist of two-channel sensors shown in Figure 1. In the channel-1 sensor, a WSF (BCF-92) is used to increase the collection efficiency of the scintillation emitted from the scintillator and a square-type POF (BCF-98, Saint-Gobain Ceramic and Plastics) is used to transmit the light output of the WSF to a long-length POF for transmission. To clarify the collection efficiency of the scintillation by using the WSF, in this study, the scintillation yields of the UTFOD were obtained with and without the WSF, respectively. As the result, by using the WSF, the collected scintillation yield increased up to 26%. As a reference sensor to remove Cerenkov radiation, the channel-2 sensor has the same structure as that of the channel-1 sensor, but the WSF is covered with black acrylic paint to shield it from the scintillation generated by BCF-12.

The scintillations of the UTFODs are measured with a photomultiplier tube (PMT) module (H9306-03, Hamamatsu Photonics). A PMT is commonly used to measure feeble scintillation signals due to the high internal gain with the reasonable quantum efficiency. The measureable wavelength range of the PMT is from 185 nm to 900 nm, and the most sensitive wavelength is about 450 nm. At the control voltage of + 1.0 V, the typical and maximum dark currents of the PMT are about 2.0 and 10 nA, respectively. To operate the PMT, a power supply (C7169, Hamamatsu Photonics) is employed. The output voltage of the power supply is ±15 V, and the maximum output currents are 0.3 A and 0.2 A at +15 V and −15 V, respectively. A pre-amplifier (C7319, Hamamatsu Photonics) is also used to convert the current to the voltage. This type of pre-amplifier has variable conversion ratios (0.1, 1.0 and 10 V/μA) and bandwidths (20 and 200 kHz).

Throughout this study, the gamma-ray beams emitted from a Co-60 therapy unit (Theratron-780, AECL) were exploited. Although the clinical linear accelerator (CLINAC) is more clinically relevant, the Co-60 therapy unit can also be useful for the feasibility study of UTFOD due to continuous and stable gamma-ray beams. A half-life of a Co-60 is 5.271 years and energies of gamma-rays are 1.173 and 1.332 MeV. The activity of the Co-60 used in our experiments is about 3000 Ci. The field sizes of the gamma-ray beams were 5 × 5, 10 × 10, 15 × 15, and 20 × 20 cm^2^, and the source to surface distance (SSD) was 80 cm.

**Figure 2:**
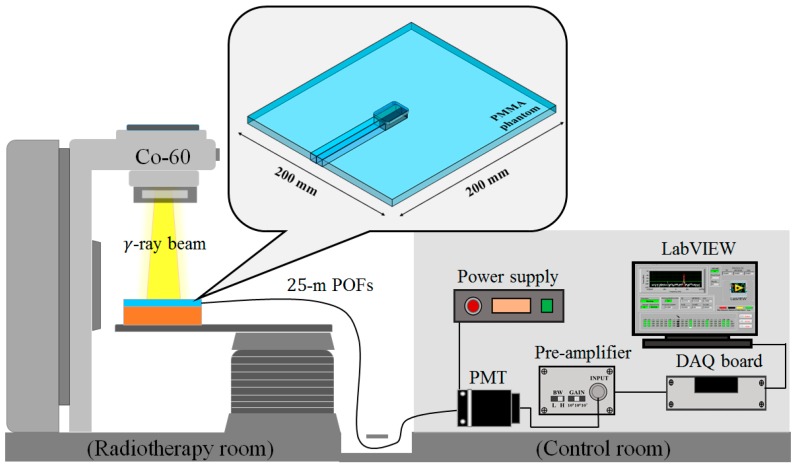
Experimental setup for measuring surface and build-up doses using the UTFODs.

Figure 2 shows the experimental setup for measuring surface and build-up doses using the UTFODs. When the UTFODs are irradiated by gamma-ray beams emitted from the Co-60 machine, scintillation signals are transmitted to the PMT by 25-m-length POFs. The output currents of the PMT are amplified and converted to voltage signals by an amplifier, and then, the voltage signals are acquired by a DAQ device (NI USB-6008, National Instruments). Finally, the acquired signals were displayed and saved with the LabVIEW program (LabVIEW 2014 SP1, National Instruments).

## 3. Experimental Results and Discussion

**Figure 3:**
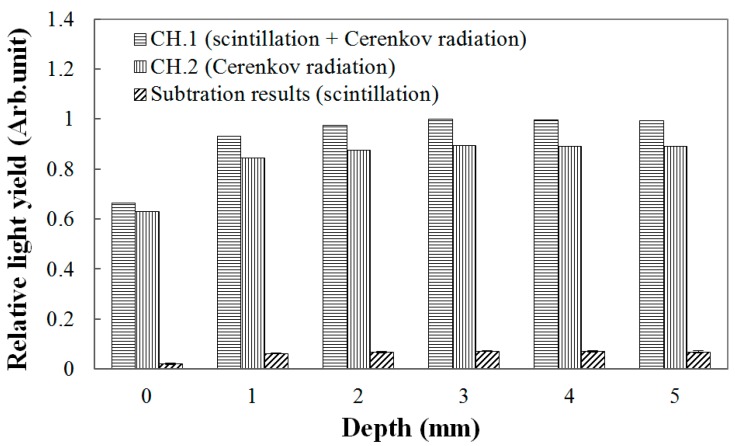
Measurement of light outputs generated from the 150-μm UTFOD according to the depths of the polymethyl methacrylate (PMMA) phantoms.

Throughout this study, the scintillation signals were obtained by subtracting the signals of the channel-2 sensors from those of the channel-1 sensors. The result in Figure 3 is an example of obtaining PDDs using the 150-μm UTFOD in a build-up region for the Co-60 gamma-ray beams. In general, Cerenkov radiation, which is known as the noise signal of FODs, is generated in the optical fibers, and its intensity varies according to the field sizes and incident angles of the radiation beams [16,17,18]. In radiotherapy dosimetry using the FODs, therefore, this type of radiation should be removed to measure only the scintillation signal [7]. As can be seen in Figure 3, because the thicknesses of the scintillators used in this experiment are very small, the Cerenkov radiation generated in the WSFs and POFs occupies greater portions of the light outputs in the channel-1 sensors. In our experiments, by using the signals of the channel-2 sensors including the black shielded WSFs, the Cerenkov radiation generated in the channel-1 sensors was discriminated, and only the scintillation signals generated from the ultra-thin organic scintillators were obtained successfully.

**Figure 4:**
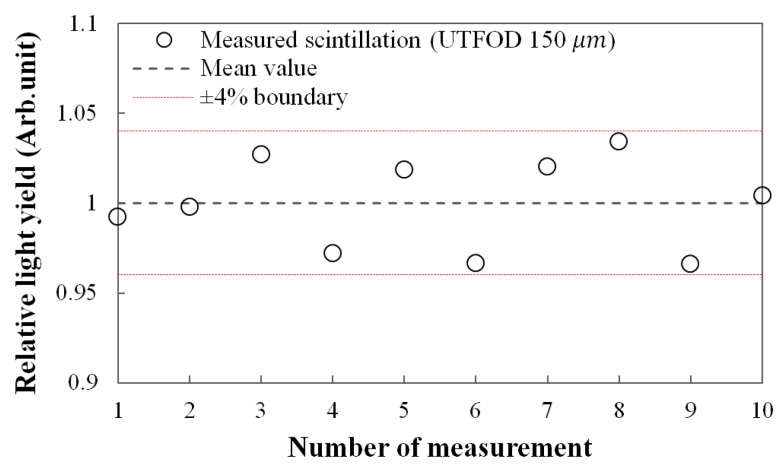
Measurement of reproducibility for the scintillation outputs of 150-μm UTFOD.

Figure 4 shows the reproducibility for the scintillation yields of UTFOD which were obtained by using the subtraction method as addressed in the result of Figure 3. In this experiment, the 150-μm UTFOD was exposed with 60 cGy irradiation of the gamma-rays generated from the Co-60 machine. All obtained scintillation yields were included within ±4% boundary as shown in Figure 4. The relative standard deviation for the measured scintillation yields was about 2.5%.

**Figure 5:**
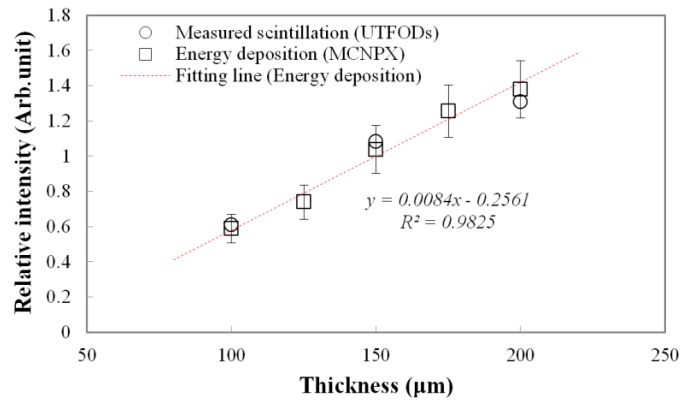
Measured scintillation yields and calculated energy depositions (using Monte Carlo N-Particle eXtended (MCNPX) code) as a function of the scintillator thickness.

Measured scintillation yields and calculated energy depositions as a function of scintillator thickness at the surface are shown in Figure 5. Typically, because the interactions of gamma-rays increase with the thickness of the medium, gamma-rays induced energy deposition on the medium grows with the thickness. Therefore, a thicker scintillator emits more scintillation. To estimate the scintillation outputs according to the thicknesses, the energy depositions on the PS, which is a main component of the scintillator, were calculated by using the Monte Carlo N-Particle eXtended (MCNPX) simulation. As a result, the energy depositions linearly increased according to the thicknesses. In our experiment, as expected, the scintillation outputs as a function of the thickness also increased with a nearly-linear trend, but had some differences from the calculated energy depositions; here, the differences grew with the thickness at about 3.75%, 4.22%, and 5.11% for the 100, 150, and 200 μm thicknesses, respectively. Although optical quenching, which occurs by the self-absorption of non-scintillation components, can increase with the scintillator thickness, this type of quenching is negligible for thin scintillators [19]. These differences are mainly induced by a small discrepancy in the scintillator thickness.

**Figure 6:**
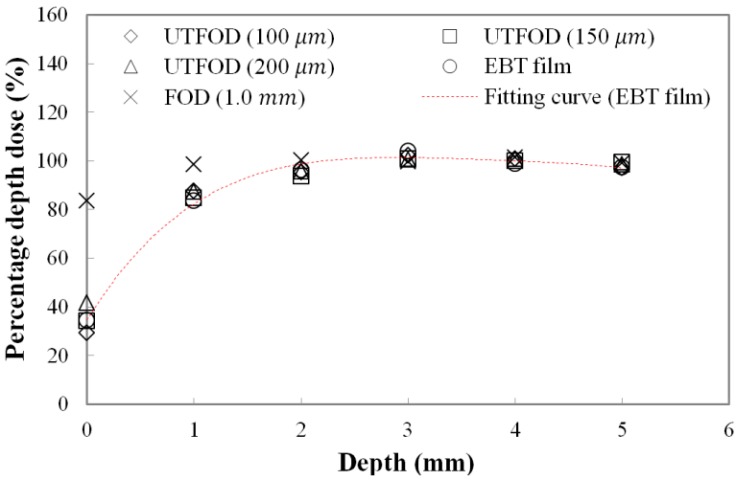
Measured percentage depth doses (PDDs) using the UTFODs in a build-up region for gamma-ray beams generated from a Co-60 therapy machine.

Measured PDDs at the surface and build-up regions for the gamma-ray beams using UTFODs are shown in Figure 6. In radiotherapy dosimetry, generally, the dose distribution at the central axis is represented by the PDD. The PDD is the percentage of the absorbed dose at a certain depth (*d_t_*) to the maximum absorbed dose at a reference depth (*d_max_*) as follows [20]:

(1) $$\text{PDD}(\%)=\frac{d_{t}}{d_{max}}\times 100$$

The PDD can vary with the irradiation conditions such as SSD, beam energy, and field size. In case of gamma-ray beams generated from the Co-60 therapy machine, PDDs increase precipitously to the depth of *d_max_* and then, decrease gently. For the gamma rays generated from a Co-60 therapy machine, the typical maximum dose depth in a water phantom is about 5.0 mm at a 10 × 10 cm^2^ field with 80 cm SSD [20]. In this experiment, the PDDs in the build-up region (from 0 mm to 5 mm) were obtained using the UTFODs. Additionally, the PDDs were obtained with EBT films and a FOD for comparison with those of the UTFODs. The EBT films used in our experiment were GAFCHROMIC EBT3 radiochromic dosimetry films which consist of a 26–28 μm active layer between two, 120 μm transparent polyester substrates. The FOD had the same structure with that of the UTFOD, but the thickness of the scintillator was 1.0 mm. In Figure 6, at the near surface region, the PDDs obtained with the conventional FOD had significant differences from the results with the EBT films due to its relatively large sensing volume. On the other hand, the PDDs from the UTFODs are in good agreement with those from the EBT films; here, the mean differences between the PDDs of the UTFODs with 100, 150, and 200 μm thick scintillators and those of the EBT films were about 2.3%, 1.7%, and 3.0%, respectively.

**Figure 7:**
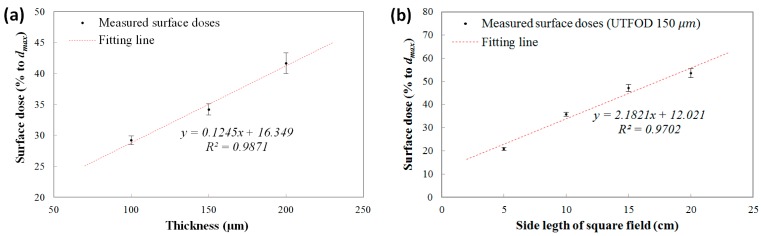
Measured surface doses according to (**a**) scintillator thicknesses; and (**b**) field sizes of the Co-60 gamma-ray beams.

The measured surface doses (% to *d_max_*) using the UTFODs are shown in Figure 7a. In the results, the surface doses increased almost linearly according to the scintillator thicknesses at about 29.2%, 34.2%, and 41.7% for the 100, 150, and 200 μm thicknesses, respectively. In the near-surface regions for the therapeutic photon beams such as high-energy *X* rays and gamma rays, the PDDs increased steeply with a nearly linear trend. The surface doses obtained with the UTFODs can be regarded as averaged PDDs over each scintillator thickness (100, 150, and 200 μm); therefore, the surface doses using the UTFODs grew linearly with the scintillator thickness. In our experimental results, meanwhile, the result for the measured surface dose using the UTFOD with a 150 μm thick scintillator matched well with the result for the EBT films (34.5%) shown in Figure 6. In addition, the center depth of the 150-μm scintillator is about 75 μm and is very close to the recommended measurement depth of skin dose; as mentioned above, the measurement of the skin dose is recommended at a depth of 70 μm [15]. Therefore, we used the UTFOD incorporating a 150 μm thick scintillator in the following experiments.

The measured surface doses according to the irradiation field sizes of the Co-60 gamma-ray beams using the UTFOD incorporating a 150 μm thick scintillator are shown in Figure 7b. In general, the scattering of gamma photons with the collimator and the phantom increases as the field size increases [21]. As a result, the scattered low-energy gamma photons and electrons induced surface dose grows with the field size. In our experiment, the surface doses obtained with the UTFOD increased with a nearly linear trend according to the field size.

**Figure 8:**
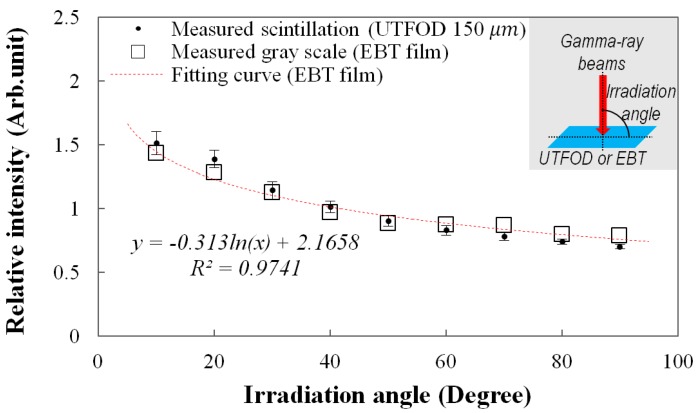
Measurement of angular response of the UTFOD.

The angular response of the UTFOD can be found in Figure 8. In this experiment, the scintillation yield of the UTFOD was measured according to the irradiation angle of the gamma-ray beams. As the result, the scintillation yield of the UTFOD decreased with the irradiation angle of the gamma-ray beams. This phenomenon can be induced by penetration paths of the gamma photons within a medium. Although the thickness of the scintillator incorporated in the UTFOD is very small, the penetration path length of the gamma photon increases with decrease of the irradiation angle. In general, the energy deposition of the gamma photon increases according to its path length therefore the scintillation yield of the UTFOD grows with decreasing the irradiation angle of the gamma-ray beams. In this experiment, the angular response of the EBT films was also measured to compare with that of the UTFOD. The results of the EBT films decreased logarithmically according to the irradiation angles and were in good agreement with those of the UTFOD.

**Figure 9:**
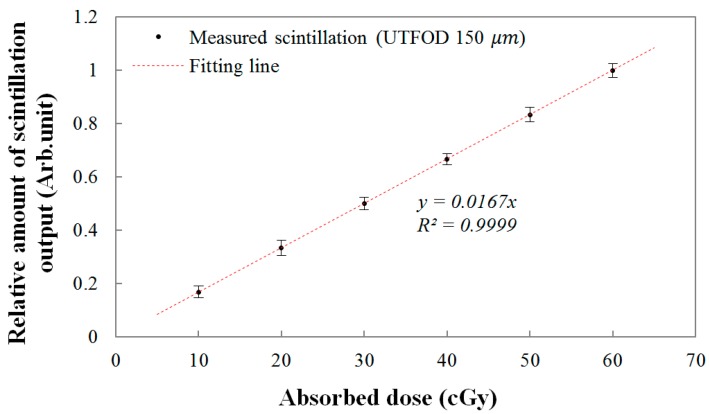
Linear response of the UTFOD according to the absorbed doses.

Finally, the linear response of the UTFOD was evaluated as a function of the absorbed dose. The relationship between the scintillation amount from the UTFOD and the absorbed doses are shown in Figure 9. In this experiment, the absorbed doses were determined by the irradiation time of the gamma-ray beams generated by the Co-60 therapy machine. As the absorbed dose increased, the scintillation amount increased linearly according to the linear fitting equation with a R-square value of 0.9999. In general, the R-square value, which indicates the accuracy of the correlation between the obtained data and the fitting model, varies from 0 to 1; here, “1” means that the fitting model is perfectly fitted with the data.

## 4. Conclusions

In radiation treatment, the accurate measurement of the skin dose is very difficult due to the limitations of conventional dosimeters. In this study, to measure the skin dose, we proposed an innovative method using UTFODs. These types of dosimeters cannot only be used to measure the skin or surface doses but also provide depth dose measurements with high spatial resolution.

As a feasibility study on UTFODs measuring skin doses with practical radiation treatment, prototype UTFODs were fabricated in this study using ultra-thin organic scintillators, WSFs, and POFs. In order to eliminate the Cerenkov radiation generated in the POFs, the UTFODs consisted of two-channel sensors, and the difference between the two sensors, which represents only the scintillation signal, was obtained with the subtraction method. By using the UTFODs, the PDDs from the surface to the maximum dose depth were obtained. Especially, the surface doses were obtained as functions of the scintillator thicknesses and the irradiation field sizes of the gamma-ray beams. From the results, the PDDs obtained with the UTFODs are in good agreement with those obtained with the EBT films, and the surface doses linearly increased with the scintillator thicknesses and the irradiation field sizes. Additionally, the scintillation outputs of the UTFODs had a linear trend to the absorbed doses.

Further study will be carried out to reduce the total thickness of the UTFODs. Although the sensor probes of the UTFODs used in this study have thicknesses of few hundred micrometers, the total thicknesses of the UTFODs are too large for application in practical radiation treatment due to the thickness of the WSFs; here, the WSFs used in this study have a thickness of 1.0 mm. Therefore, thinner WSFs should be considered in future UTFODs. In addition, throughout this study, the results obtained by the EBT films were used to compare with those by the UTFODs. Although the EBT films are widely used in radiotherapy dosimetry, results from a well-characterized parallel plate chamber would constitute the practical ground truth. Therefore, the comparison study using the parallel plate chamber should also be conducted. It is anticipated that the UTFOD proposed in this study can effectively be used to measure skin and relative depth doses in radiotherapy dosimetry.
